# Malaria with neurological involvement in Ugandan children: effect on cognitive ability, academic achievement and behaviour

**DOI:** 10.1186/1475-2875-10-334

**Published:** 2011-11-03

**Authors:** Paul Bangirana, Seggane Musisi, Michael J Boivin, Anna Ehnvall, Chandy C John, Tracy L Bergemann, Peter Allebeck

**Affiliations:** 1 Department of Psychiatry, Makerere University College of Health Sciences, Kampala, Uganda; 2 Department of Public Health Sciences, Karolinska Institutet, Stockholm, Sweden; 3 Departments of Psychiatry and Neurology/Ophthalmology, Michigan State University, East Lansing, MI, USA; 4 Neuropsychology Section, Department of Psychiatry, University of Michigan, Ann Arbor, MI, USA; 5 Institute of Clinical Neurosciences, Department of Psychiatry, Gothenburg University, Gothenburg, Sweden; 6 Psychiatric Clinic of Varberg, Halland County Council, Sweden; 7 Department of Pediatrics, University of Minnesota, Minneapolis, MN, USA; 8 Division of Biostatistics, School of Public Health, University of Minnesota, Minneapolis, Minnesota, USA

**Keywords:** malaria, cognition, behaviour, academic achievement, neuropsychology

## Abstract

**Background:**

Malaria is a leading cause of ill health and neuro-disability in children in sub-Saharan Africa. Impaired cognition is a common outcome of malaria with neurological involvement. There is also a possibility that academic achievement may be affected by malaria with neurological involvement given the association between cognitive ability and academic achievement. This study investigated the effect of malaria with neurological involvement on cognitive ability, behaviour and academic achievement.

**Methods:**

This prospective case-control study was carried out in Kampala City, Uganda between February 2008 and October 2010. Sixty-two children with a history of malaria with neurological involvement were followed up and given assessments for cognitive ability (working memory, reasoning, learning, visual spatial skills and attention), behaviour (internalizing and externalizing problems) and academic achievement (arithmetic, spelling and reading) three months after the illness. Sixty-one community controls recruited from the homes or neighbouring families of the cases were also given the same assessments. Tests scores of the two groups were compared using analysis of covariance with age, sex, level of education, nutritional status and quality of the home environment as covariates. This study was approved by the relevant ethical bodies and informed consent sought from the caregivers.

**Results:**

Children in the malaria group had more behavioural problems than the community controls for internalizing problems (estimated mean difference = -3.71, 95% confidence interval (CI), = -6.34 to -1.08, p = 0.007). There was marginal evidence of lower attention scores (0.40, CI = -0.05 to 0.86, p = 0.09). However, excluding one child from the analyses who was unable to perform the tests affected the attention scores to borderline significance (0.32, CI, = 0.01 to 0.62, p = 0.05). No significant differences were observed in other cognitive abilities or in academic achievement scores.

**Conclusion:**

Malaria with neurological involvement affects behaviour, with a minimal effect on attention but no detectable effect on academic achievement at three months post discharge. This study provides evidence that development of cognitive deficits after malaria with neurological involvement could be gradual with less effect observed in the short term compared to the long term.

## Background

Malaria, caused by *Plasmodium falciparum*, is one of the most commonly occurring infections in children in sub-Saharan Africa with approximately 515 million clinical episodes annually [1]. It is a leading cause of ill health, neuro-disability and death in the tropics [2]. One of its most severe forms is cerebral malaria which has a mortality of about 20% [3]. In addition to the high mortality associated with cerebral malaria, studies in African children have documented a high rate of cognitive deficits that may persist up to eight years after the illness [4]. Two prospective studies in Ugandan children with a history of cerebral malaria found that 21% had cognitive impairment at six months and 26% at 24 months post discharge [5,6]. Impairments were observed mainly in attention and working memory. In both studies, cerebral malaria was associated with a 3.7-fold risk of cognitive impairment compared to community control children.

In a Kenyan study, malaria with impaired consciousness was associated with deficits in attention at three years post illness with 14% impaired [7]. A similar frequency of impairment has been observed by Kihara and colleagues in Kenyan children using event-related potentials as opposed to the conventional paper-pencil neuropsychological tests [8]. In Kihara's study, children with severe malaria had slower responses to visual and auditory cues than the controls.

Carter *et al *also observed that both cerebral malaria and malaria with complicated seizures led to deficits in non-verbal functioning and speech and language in Kenyan children [4]. These children, tested nine years post illness, had 24% cognitively impaired. Earlier studies in Senegalese and Ghanaian children also showed impaired cognition after cerebral malaria [9]. Cerebral malaria, malaria with seizures and malaria with impaired consciousness, all shown to lead to cognitive deficits in African children, have been collectively termed 'malaria with neurological involvement' [10].

Working memory and attention deficits, which are commonly reported outcomes in the above studies, have been associated with poor reading and arithmetic achievement in children [11-19] implying a possible effect of malaria with neurological involvement on academic achievement. Working memory is needed in arithmetic for multi-digit calculations, activating, retrieval and manipulating information in long-term memory [13,20]. Reading ability also depends on working memory for decoding unfamiliar words, retrieving semantic knowledge of familiar words, recalling previously read text, and anticipating where the passage is going [21]. It is reasonable to expect that children with impaired working memory will be educationally disadvantaged and experience a range of learning difficulties [22].

Lower grades in school have also been reported among children exposed to infection with non-severe malaria [23,24] suggesting that malaria with neurological involvement is likely to result in similar or worse outcomes. Two large intervention studies in African children showed improved school performance in a group of children that received malaria prophylaxis compared to a control group [25,26]. It can be concluded from these studies that malaria occurrence may affect school performance. Despite this plausible link of malaria affecting academic functioning, no studies have directly examined the effect of malaria with neurological involvement on academic functioning.

There is also inconclusive evidence on whether malaria with neurological involvement may result in dysfunctional behaviour in children. Studies that have assessed behavioural outcomes of cerebral malaria were either case studies [27,28] with limited ability to generalize, do not describe the behavioural functions affected [4,7], have not used standardized behavioural tests [29] or used foreign norms for comparison [30]. Documentation of the behavioural problems after malaria with neurological involvement can highlight problems that need to be assessed in survivors and help in development of appropriate interventions for affected children [30].

This study assessed cognition, behaviour and academic achievement in Ugandan children three months after an episode of malaria with neurological involvement.

## Methods

### Study population and recruitment

Children were recruited from Mulago Hospital, the National Referral Hospital of Uganda, and from Nsambya, Rubaga and Mengo Hospitals, all located in Kampala, Uganda's capital city. The latter three are large private mission hospitals. Participants were children aged five to 12 years presenting with malaria (*Plasmodium falciparum *on blood smears) and either one or more of the following; 1) convulsive seizures lasting over 15 minutes or repeated seizures observed by the parent or during admission at the hospital, 2) impaired consciousness (Glasgow coma scale score of 14 and below), or 3) coma (i.e., un-arousable coma with normal cerebrospinal fluid). A physical examination and medical history were done on admission.

At discharge, home directions and telephone contacts were obtained from the parents/caregivers and an appointment made for the baseline assessment three months later. In the interim period, a home visit was made to assess the quality of the home environment and to recruit a control child. Inclusion criteria for the control children were: aged between five to 12 years and not ill at the time. The control child was required to come to Mulago Hospital with the case for the baseline assessments at three months. The exclusion criteria for both groups were: history of or present meningitis, encephalitis, other central nervous system infections, sickle cell disease, epilepsy, multiple seizures, developmental delay or hospitalization for malnutrition.

Ninety children with malaria were recruited for the study. Of these, 10 did not meet the above inclusion criteria and were excluded and one died during admission, leaving 79 children for follow-up. By the three-months hospital visit, 17 children had either withdrawn from the study or had been lost to follow-up, leaving 62 children who were given assessments of cognition, behaviour and academic skills. These 62 children included nine with cerebral malaria, 34 having malaria with seizures and 19 having malaria with impaired consciousness. One of the nine children with cerebral malaria had severe neurologic impairment from the disease and could not do the tests. The lowest possible cognitive test scores were assigned to this child.

Sixty-one community control children were recruited from the homes or neighbourhoods of the cases with malaria. They first underwent a physical examination and medical history to make sure they were healthy and had no prior cerebral insult before undergoing the same assessments as the malaria group.

Written informed consent was obtained from the parents or guardians of study participants and assent from children aged seven years and older. Ethical approval for this study was granted by the Institutional Review Board for Human Studies at Makerere University College of Health Sciences and the Uganda National Council for Science and Technology.

### Assessments

#### Kaufman assessment battery for children second edition (KABC-II)

The KABC-II [31] is a comprehensive assessment of cognitive ability containing a number of critical scales that have been adapted, piloted and validated in malaria studies among African children in Kenya [7], Senegal [32] and Uganda [5]. It retains its construct validity when used in Ugandan children to access working memory, visual spatial ability, learning, and reasoning [33]. In the Ugandan validation study, test items from the Gestalt Closure subscale found to be inappropriate were omitted. In addition, the subscales testing Knowledge (Crystallized Ability) were not included in this validation as they were not culturally appropriate. The KABC maintained its construct validity in this validation study. Only the test items used in this validation study were used in the present study. The primary outcome measure in this test was working memory. The secondary outcomes were visual spatial ability, learning and reasoning.

#### Test of variables of attention (TOVA)

The TOVA is a computer-administered continuous performance test used in the diagnosis and monitoring of children and adults with attention deficit disorders [34]. The TOVA has been used in previous malaria studies in Senegal and Uganda [5,6,32]. It is a sensitive measure of cerebral insult from malaria as indicated by the persisting attention deficits at 24 months in Ugandan children with cerebral malaria [6]. Being a computerized test with no cultural bias in the test stimuli and given its consistent sensitivity to the effects of malaria on the brain in previous studies, no changes were made to this test for use in the present study. The primary outcome measure was the signal detection D prime score, which is a measure of overall attention capacity.

#### Child behaviour checklist (CBCL)

The CBCL is a paper-pencil child behavioural rating scale consisting of 120 items to which a parent/guardian responds [35]. It has been validated in 30 societies, has proven useful in multi-cultural assessment of children [36] and has fair reliability in Ugandan children after adaptation that involved back translation of the test items [30]. The test-retest and internal reliabilities of the CBCL in Ugandan children were between 0.64 and 0.83 [30]. The main outcome scores were internalizing problems and externalizing problems.

#### Wide range achievement test-third edition (WRAT-3)

The WRAT-3 is a measure of the codes needed to learn the basic skills of reading, spelling and arithmetic [37,38]. It has been used earlier for research in Ugandan children with HIV [39], but no formal adaptation has been done for this test. Scores for reading, spelling and arithmetic were used to determine the child's academic achievement in those domains. Arithmetic and reading were the primary outcomes and spelling the secondary outcome.

#### Middle childhood home observation for the measurement of the environment (MC-HOME)

The MC-HOME identifies parental behaviours that are important to children's cognitive development and academic skills [40]. The MC-HOME, as adapted to the Ugandan setting by Boivin and colleagues [5], was used in this study. It has 58 items measuring the amount of stimulation and learning opportunities available to the child in the home, which were summed into a single score. This score indicates the quality of the home environment, which is predictive of working memory performance in Ugandan children [41].

#### Data analysis

Data was analysed using SPSS 17.0. Analysis of covariance was used to compare cognitive ability, academic achievement and behaviour between the malaria group and the control group. The child's age, level of education, MC-HOME score, weight for age z score, and sex were entered as covariates in the model. The Benjamini-Hochberg correction was used to account for multiple comparisons [42].

## Results

Community control children were older and weighed more on average than the children in the malaria group (Table 1). Other demographic characteristics were similar in the two groups. At the univariate level, the malaria group had poorer outcomes than the control group (Table 2). In the ANCOVA model, children in the malaria group had more behavioural problems than the community controls for internalizing problems (estimated mean difference = -3.71, confidence interval (CI) = -6.34 to -1.08, p = 0.007). There were no significant differences in any of the other cognitive or academic achievement measures between the groups including attention, the primary outcome (0.40, 95%, (CI) = -0.05 to 0.86, p = 0.09) (Table 3). However when the child with severe neurologic impairment was excluded from these analyses, a borderline significant difference between the groups was seen in attention (0.32, CI, = 0.01 to 0.62, p = 0.05).

**Table 1:** Participants' demographic characteristics

Domain	Malaria group	Control group	P
Gender, male N (%)	37 (59.7)	34 (55.7)	0.40
Age (years)	7.22 (1.69)	8.07 (2.01)	0.01
School grade	2.41 (1.54)	2.84 (1.87)	0.17
Weight (kgs)	21.34 (5.22)	24.18 (6.29)	0.008
Height (cm)	119.22 (14.52)	120.87 (12.11)	0.53
Weight for age z score	-0.96 (1.14)	-0.74 (1.25)	0.30
Home environment score	23.46 (6.22)	23.93 (4.95)	0.64
Socio-economic score	10.26 (3.80)	9.38 (3.16)	0.19

cm = centimetres, kgs = kilograms, N = Number. All values are Mean (SD) unless otherwise stated.

**Table 2:** Descriptive statistics of the test scores

	Total sample		Malaria group	Control group	
**Variable name**	**M (SD)**	**Range**	**M (SD)**	**M (SD)**	**P**

Working Memory	26.96 (7.41)	0.00 to 43	25.29 (7.95)	28.66 (6.44)	0.01
Visual Spatial ability	31.56 (14.82)	0.00 to 79	29.10 (13.90)	34.07 (15.42)	0.06
Planning	7.32 (5.19)	0.00 to 31	6.61 (4.81)	8.03 (5.50)	0.13
Learning	89.41 (41.28)	0.00 to 201	81.84 (40.86)	97.10 (40.59)	0.04
Attention	2.03 (1.39)	-8.53 to 4.42	1.68 (1.63)	2.40 (0.97)	0.00
Reading	14.55 (9.84)	0.00 to 43	12.59 (9.45)	16.68 (9.89)	0.02
Spelling	13.46 (8.90)	0.00 to 34	12.16 (8.96)	14.88 (8.69)	0.10
Arithmetic	13.98 (7.98)	0.00 to 33	11.97 (7.57)	16.14 (7.92)	0.00
Internalizing Problems	11.97 (7.33)	0.00 to 43	13.41 (7.91)	10.52 (6.60)	0.03
Externalizing Problems	17.17 (10.04)	3.00 to 50	18.16 (10.09)	16.18 (9.97)	0.28

**Table 3:** Estimated mean differences in test scores between the community controls and malaria group

Specific ability	Estimated mean differences (confidence interval)	P^1^
Attention	0.40 (-0.05, 0.86)	0.09
Working Memory	1.53 (-0.52, 3.58)	0.15
Visual Spatial ability	-0.25 (-3.72, 4.23)	0.90
Reasoning	0.23 (-1.17, 1.63)	0.75
Learning	4.35 (-6.13, 14.84)	0.42
Arithmetic	1.41 (-0.36, 3.17)	0.12
Reading	1.21 (-0.96, 3.38)	0.28
Spelling	0.40 (-1.91, 2.70)	0.74
Internalizing Behaviour	-3.71 (-6.34, -1.08)	0.007
Externalizing Behaviour	-1.60 (-5.29, 2.09)	0.40

^1^ANCOVA with covariates; child's age, level of education, quality of home environment score, weight for age z score and sex

With the sample size of 61 children with malaria, the study had sufficient power (at least 80%) to detect differences of 0.71 in attention, 3.76 in working memory, 4.05 in arithmetic, 4.99 in reading, 3.75 in internalizing problems and 5.13 in externalizing problems.

## Discussion

This study set out to examine in children, the effect of malaria with neurological involvement on cognitive ability, academic achievement and behaviour three months after infection. Malaria with neurological involvement was associated with increased behavioural problems and a minimal effect on attention but did not affect academic functioning.

This is the first study to use standardized measures to assess both the behavioural and academic outcomes after malaria with neurological involvement. Recent studies from Uganda show that malaria with neurological involvement is associated with severe behavioural problems like hyperactivity, aggressive behaviour, autism spectrum disorders, internalizing problems (withdrawn/depressed and thought problems) and externalizing problems (aggressive behaviour and oppositional defiant behaviour) [29,30]. The current study did show an increase in internalizing problems as previously shown [30] and showed a modest but non-significant increase in externalizing problems similar to those reported by Idro *et al *[29] in a clinic-based case-control study. The difference in the externalizing problems results across the studies may be due to the assessment methods used in the two earlier studies compared to the current one. One study [30] used the CBCL but did not use Ugandan norms to determine behavioural problems, while Idro *et al *[29] reviewed hospital records that may not have captured the same information for all patients. In addition, the children in Idro *et al *[29] were from a specialist child neurology clinic which treats patients with severe behavioural problems. This may explain why Idro and colleagues [29] report severe overt behavioural problems while this study, and a previous one [30] using the CBCL, both report internalizing problems that are covert in nature. The present prospective study is best suited to assess behavioural outcomes after severe malaria than a cross-sectional study despite the small sample size and loss to follow-up.

Both retrospective and prospective studies have shown that severe malaria does affect cognition with rates of impaired cognition ranging between 14 to 26% [4-8,32]. The prospective studies from Uganda especially show an increasing trend of cognitive deficits from 21% at six months to 26% at 24 months [5,6]. However it is the assessments at discharge and three months follow-up in these studies that may explain the present study's finding of minimal effect on cognition. At discharge, 36% of the children with cerebral malaria were impaired compared to 11% of the controls, but this rate fell to 19% *vs *7% at three months [5]. What is more important is that these differences in impairment were significant at discharge but not at three months. Important to note is these two Ugandan studies used combined cognitive test scores to determine children who were impaired while the present study is assessing individual cognitive skills.

This implies that much as malaria with neurological impairment has been associated with cognitive impairment, there seems to be a gradual development of these deficits, which become more pronounced and severe, as was shown at the 24-months assessments by John *et al *[6]. This may explain why minimal effect was seen on cognition and no effect on academic achievement in the current study done at three months follow-up. The high frequency of impairment at discharge in the Boivin *et al *[5] study could therefore be attributed to the malaise associated with cerebral malaria illness and not necessarily the effect of the disease on the brain; otherwise the frequency of impairment would not drop from 36% at discharge to 19% at three months and then increase to 21% at six and 26% at 24 months, as was observed.

In a re-analysis of cognitive test scores from John *et al *[6] and Boivin *et al *[5] using a novel method employing a global normalized Z-score [Bergemann TL, Bangirana P, Bruno G, Boivin MJ, Connett JE, John CC: Statistical approaches to assess the effects of disease on neurocognitive function over time, Submitted], more differences were observed in test scores between the malaria group and the community controls than were seen in the earlier two studies. Though significant differences were observed at zero, three, six and 24 months, the effect sizes were smallest at three months with a p value of 0.04 compared to the other time points with very small p values and large effect sizes. This too supports the conclusion that cognitive deficits after severe malaria are not evident at three months after the illness.

The heterogeneous nature of the malaria group may be another reason why minimal group-average effects were seen in the current study. Of the sixty-two children with malaria in this study, nine had cerebral malaria, 34 had seizures, and 19 had impaired consciousness but no coma. The study by Boivin *et al *[5], which assessed cognition at three months, included only children with cerebral malaria, the most severe form of malaria. Given the less severe effects of cerebral malaria on cognition seen at three months in Boivin *et al *[5] and Bergemann and colleagues [Bergemann TL, Bangirana P, Bruno G, Boivin MJ, Connett JE, John CC: Statistical approaches to assess the effects of disease on neurocognitive function over time, Submitted], compared to the other time points, it was unlikely that the present study, using similar assessments and having more children with the less severe forms of malaria, would show cognitive deficits at the same time point.

Similarly, absence of an effect on the academic achievement scores could also be attributed to the heterogeneous sample and assessment at three months, which is too early to see any effect. This contrasts with studies in which effects on academic performance were seen at two weeks after infection with less severe forms of malaria [43]. Other studies have also observed effects on academic performance after infection with less severe malaria [23,24]. One difference between this study and the above studies is that they used measures of academic performance developed in the country or specific region while assessment of academic achievement in this study was developed in the west. It is likely that the WRAT-3 in this study is not a sensitive measure of academic achievement in the early period after malaria infection. These contrasting results call for further assessment of school performance after severe malaria in Ugandan children using both local and western tests.

This study adds to the body of knowledge that malaria with neurological involvement does affect behaviour and cognition, highlighting the need for intervention among child survivors. The observation that these deficits may become more evident with time also necessitates interventions to arrest this trend. It has been previously shown that computerized cognitive rehabilitation training does improve cognition and behaviour after severe malaria infection [44,45].

However, there are several limitations in this study that necessitate caution when interpreting the results. The case-control design used is not sufficient to test causality especially with the limited follow-up time and demographic differences in the cases and controls. The observed effects could also include artefacts of these differences. The heterogeneous sample of the malaria group may affect outcomes since risk factors for cognitive deficits like coma, seizures, neurological signs, hypoglycaemia and malnutrition [3,5,6,32,46] may be present in some forms of malaria and not others. This is more significant when the numbers of children with these different malaria forms are not similar in the sample. In addition, diagnosis of cerebral malaria did not include retinopathy examination which increases the specificity in cerebral malaria diagnosis [47].

## Conclusions

Malaria with neurological involvement results in behaviour problems, affects cognition minimally but may not affect academic achievement at three months. Understanding the burden of malaria with neurological involvement on cognition, academic achievement and behaviour can best be achieved with large long-term prospective studies. Despite the minimal effects in this study, interventions planned early may improve the lives of these children.

## Competing interests

The authors declare that they have no competing interests.

## Authors' contributions

PB conceived the study, participated in the data collection, analysis, interpretation and writing of the manuscript. PA, MJB, CCJ, AE and SM participated in the interpretation and writing of the manuscript. TLB participated in the analysis, interpretation and writing of the manuscript. All authors read and gave approval of the final version to be published.
